# How Stereotypes Affect Pain

**DOI:** 10.1038/s41598-019-45044-y

**Published:** 2019-06-13

**Authors:** Katharina A. Schwarz, Christian Sprenger, Pablo Hidalgo, Roland Pfister, Esther K. Diekhof, Christian Büchel

**Affiliations:** 1 Department of Systems Neuroscience, University Medical Center Hamburg-Eppendorf, Hamburg, Germany; 2 Institute of Psychology, University of Würzburg, Würzburg, Germany; 3 Institute of Human Biology, University of Hamburg, Hamburg, Germany

**Keywords:** Perception, Social neuroscience

## Abstract

Stereotypes are abundant in everyday life – and whereas their influence on cognitive and motor performance is well documented, a causal role in pain processing is still elusive. Nevertheless, previous studies have implicated gender-related stereotype effects in pain perception as potential mediators partly accounting for sex effects on pain. An influence of stereotypes on pain seems indeed likely as pain measures have proven especially susceptible to expectancy effects such as placebo effects. However, so far empirical approaches to stereotype effects on pain are correlational rather than experimental. In this study, we aimed at documenting gender-related stereotypes on pain perception and processing by actively manipulating the participants’ awareness of common stereotypical expectations. We discovered that gender-related stereotypes can significantly modulate pain perception which was mirrored by activity levels in pain-associated brain areas.

## Introduction

Stereotypes are ubiquitous in our society, detailing specific expectations evoked by gender, ethnicity, nationality, religion, or sexual orientation. Their effects on the stigmatized groups can be detrimental and the effects span such diverse fields as athletics or skilled performance^1–3^, as well as various cognitive abilities, such as mathematics or verbal skills^3–6^.

A common definition states stereotypes to be “beliefs about characteristics, attributes, and behaviors of members of certain groups”^7^. In essence, therefore, stereotypes are a-priori expectations that have little to do with the individual, but rather with the specific role an individual is expected to play. In terms of gender, stereotypes about feminine and masculine behaviour are also prevalent in beliefs about prototypical gender roles. Related to pain, such gender roles, for example, include that typically men are less willing to report pain than women, or that men have higher endurance of pain^8^ (Please note, however, that such gender roles can be different across different cultures. That is, whereas in the USA where most previous studies on gender roles in pain were conducted, these gender roles might be strong, they might be less prevalent and more ambiguous in Germany, where the present study took place). But how do these stereotypical beliefs align with reality? Many studies have found differences in the response to pain between males and females in accordance with these stereotypes^9^. And, yet, a systematic review of 10 years of related literature has found no clear and consistent pattern of sex differences in human pain sensitivity^10^. Previous studies on pain perception have investigated whether the irregular and often rather inconsistent sex effects in pain measures might partly depend on such gender role expectations, or, in other words, stereotypes^11–15^. Indeed, questionnaire measures of these expectations at least partly accounted for observed sex effects in several correlational analyses, pointing to an important field in which stereotypes might have significant consequences. Yet, so far there is little conclusive evidence for a causal relation between gender-specific expectations and observed patterns in pain measures (but see^16–18^). Strong, conclusive evidence, however, is needed if gender-related expectations regarding pain measures are to be discussed as relevant contributors to actual sex differences in pain perception and processing.

The question of stereotype effects on pain perception and processing is especially important because recent accounts see a strong role of social expectancies such as stereotypes in the clinical environment, mainly in terms of negative consequences, i.e., by promoting avoidance behaviour in patients, impaired communication, and poorer adherence to treatment plans^19^. If stereotypes affect pain perception and processing, this is a clear indication that common clinical measures might be influenced even more directly by social expectancies than is currently assumed.

Such a scenario is indeed likely, as a wealth of research on expectancy effects on pain perception and processing suggests that these measures are especially susceptible to the influence of expectations. Placebo and nocebo effects on pain perception and processing are well documented in the literature, demonstrating influences on pain reports as well as on a variety of physiological measures, such as neurotransmitter release, hormonal responses, cardiovascular responses, and changes in blood oxygen level dependent (BOLD) activity in the brain and spinal cord^3,20–27^. These physiological measures provide insights regarding the processing of the pain, the physiological reaction of the body in response to the pain, and indicate the underlying mechanisms leading to altered pain perception. For example, placebo instructions led to less perceived pain as well as an increased release of endogenous opioids and changes in pain processing areas in the brain and in the spinal cord^28,29^. However, there are crucial differences between expectations underlying placebo effects and stereotypes: whereas placebo effects rely on expectations that a person generates him- or herself (due to experience or external suggestions), social expectancies and stereotypes are characterized by relying primarily on other people’s expectations. For example, some accounts postulate that stereotype threat is indeed driven by an imbalance between a person’s expectations on his or her performance and other people’s (usually negative) expectations (based on group affiliation) on that person’s performance^30^. Thus, the question of stereotype effects on pain also more generally targets whether expectations that are not generated by an individual (but of which the individual is aware) can still significantly affect that individual’s perception and processing of pain.

The main goal of the present study therefore pertains to the question of whether or not expectations based on pain-related stereotypes affect pain perception and processing. To this end, we employed behavioural and neurophysiological measures to investigate how gender-related stereotypes affect pain reports and the neurophysiological underpinnings of pain processing as measured by functional magnetic resonance imaging (fMRI)^31^. We analysed the data of 105 male participants on two days each. On day 1, we obtained basic heat pain measurements including pain sensitivity and pain threshold measures. On day 2, we manipulated the participants’ awareness of common stereotypes regarding their own pain sensitivity by subtly briefing them about alleged evolutionary effects on pain sensitivity. One group was told that, as men used to be hunters and gatherers and therefore more prone to injury, they are generally less sensitive to pain than women (*MLPS* group, n = 34). A second group was told that, as women undergo the painful process of childbirth, women are generally less sensitive to pain than men (*FLPS* group, n = 35; see S1 File). A third group did not receive any further gender-related information (*Control* group, n = 36). After the manipulation, participants underwent the same experimental paradigm as on day 1.

We hypothesized the *MLPS* group to show decreased pain sensitivity, whereas we expected the *FLPS* group to show increased pain sensitivity^28,32^. Moreover, we expected these effects to be mirrored in the neurophysiological response in pain-related brain areas^33^.

## Results

### Behavioural Results

**Figure 1:**
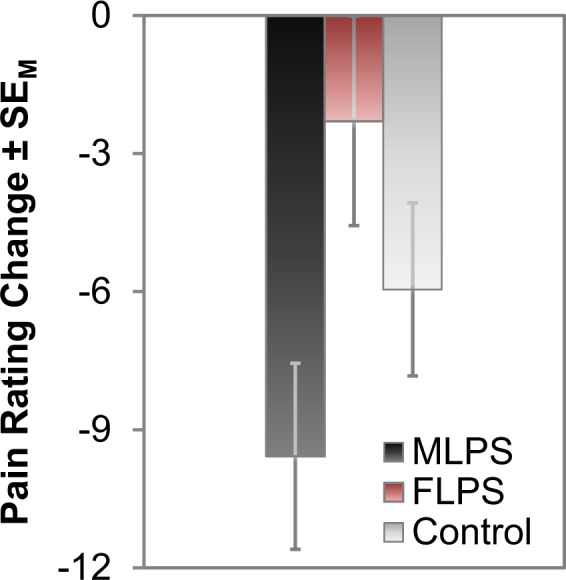
Changes in pain sensitivity ratings. Changes in pain sensitivity ratings (day 2 - day 1) for each group (raw data are shown in Supplementary Fig. S1).

Our main analysis of interest concerned the difference between the two expectancy manipulation groups, i.e., *MLPS* vs. *FLPS* (Fig. 1; Supplementary Fig. S1). The results show a significant effect of this manipulation on pain reports (interaction *Time* × *Gender Expectancy*; *F*(1,67) = 5.72, *p* = 0.020, η_p_^2^ = 0.08), with a prominent decrease in pain sensitivity for the *MLPS* group (14.7%) in contrast to the *FLPS* group (3.6%). The critical interaction was also significant when expanding the analysis to include the *Control* group, *F*(2,102) = 3.08, *p* = 0.050, η_p_^2^ = 0.06. This analysis further yielded a significant linear contrast *MLPS* > *Control* > *FLPS*, *F*(1,102) = 6.16, *p* = 0.015, η_p_^2^ = 0.06. The corresponding decrease and increase in pain sensitivity after the expectancy manipulation relative to the *Control* group could be interpreted in analogy to placebo and nocebo effects resulting in hypo- and hyperalgesia, respectively. Please note that pair-wise comparisons only revealed significant differences between the *MLPS* and *FLPS* group (as reported above), not between either expectancy manipulation group and the *Control* group (*p*s > 0.192). As we have pointed out in the Methods section, however, all analyses regarding the *Control* group should be treated with caution as certain stereotype-related beliefs (or a heterogeneous composition thereof) could also be effective in this group. The effects of the expectancy manipulation were also reflected in pain threshold measures, Time × Gender Expectancy, *F*(2,101) = 6.51, *p* = 0.002, η_p_^2^ = 0.11. The general response pattern clearly indicates that the MLPS group shows the strongest increase in pain threshold temperature on day 2 compared to day 1 (Supplementary Fig. S1B).

Please note that expectancy effects such as placebo or nocebo are often observed in response to open medical sham treatments and mostly arise after elaborate instruction and conditioning procedures, of which the latter seem most effective^28,32,34^. In contrast, we elicited changes in pain sensitivity simply by a subtle briefing on stereotypical gender role expectations, without any conditioning involved.

### fMRI Results

**Figure 2:**
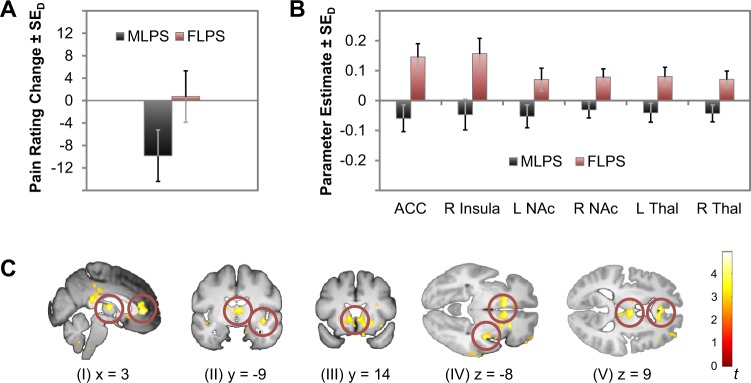
fMRI results (**A**). Behavioural measures of the participants measured by fMRI in terms of changes in pain sensitivity ratings (day 2 - day 1; scale: 0-“no pain at all”, 100-“unbearable pain”). Raw scores are plotted in Supplementary Figure S1. (**B**) Parameter estimates of peak voxels in the early pain phase for the contrast *Pain Day 2* > *Pain Day 1* in the anterior cingulate cortex (ACC; 3,38,2), right insula (41,−11,−8), bilateral nucleus accumbens (−9,14,−6/12,8,−12) and bilateral thalamus (0,−14,6/5,−9,9). (**C**) BOLD signal in the early pain phase for the contrast *FLPS(Pain Day 2* > *Pain Day 1)* > *MLPS(Pain Day 2* > *Pain Day 1)* of the ACC (I, V), the insula (II, IV), nucleus accumbens (III, IV) and thalamus (I, II, V). To better judge the extent of the activations, the display threshold is set to *p* < 0.005, 10 voxels minimum).

To ensure that the observed effects on the subjective ratings mirrored neurophysiological measures of pain processing^35^, we obtained fMRI measurements during pain stimulation in 34 participants (n_*MLPS*_ = 17, n_*FLPS*_ = 17). We focused on instruction-dependent changes in pain processing on day 2 compared to day 1. As in previous studies in which we used long pain stimulation blocks^28^, we investigated early and late pain periods separately. In the late pain phase, no significant differences were observed. During the early pain phase, however, several brain regions reflected the interaction effect of the behavioural data, including anterior cingulate cortex (ACC), right insula, bilateral nucleus accumbens and thalamus (Fig. 2; Table 1). Please note, however, that when conservatively corrected for multiple comparisons only the activation of the ACC surpassed the corrected threshold. These regions showed stronger activity on day 2 relative to day 1 in the *FLPS* group compared to the *MLPS* group and have been reported to be sensitive not only to pain in general^36,37^, but also to pain intensity, i.e., reflecting the participants’ pain experience^33,38–40^. In particular ventral striatal activation has been linked to emotional reactions to pain^41^.

**Table 1:** BOLD responses for the interaction effect *Time* × *Expectancy Manipulation* for the early pain phase in the FIR analysis; contrast: *FLPS(Pain Day 2* > *Pain Day 1)* > *MLPS(Pain Day 2* > *Pain Day 1*).

*Brain Region*	*x*	*y*	*z*	*t*	*p*_*exact*_	*p*_*corr*_
ACC	3	38	2	4.63	3.29E-05	0.044
R Insula	41	−11	−8	3.99	1.93E-04	0.180
L NAc	−9	14	−6	3.21	0.0016	0.657
R NAc	12	8	−12	3.51	2.24E-04	0.200
L Thal	0	−14	6	3.85	2.86E-04	0.239
R Thal	5	−9	9	4.07	1.59E-04	0.155

Coordinates are denoted by x, y, z in mm (MNI-space), and strength of activation is expressed in *t* values (*df* = 32). *P* values are corrected for multiple comparisons i.e. all voxels of all ROIs.

**Figure 3:**
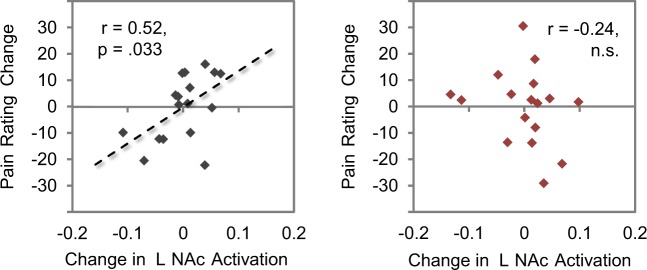
Impact of pain-related stereotypes on the dopamine system. Correlation of changes in nucleus accumbens activation (day 2–day 1) and corresponding changes in pain ratings, separately for the *MLPS* group (left panel) and for the *FLPS* group (right panel). A direct comparison of the Fisher-*Z*-transformed correlation coefficients (*Z*_*MPLS*_ = 0.57, *Z*_*FLPS*_ = −0.25) confirmed the correlations of the two groups to differ significantly, *z* = 2.17, *p* = 0.030, ε = 0.82. Both measures were centred to facilitate visual comparison of the correlations.

Moreover, the nucleus accumbens is heavily associated with the dopaminoceptive system^42^. As this system seems to be involved even before pain onset^43^, we additionally investigated the anticipation phase of the fMRI sample. Here we observed that the reduction of left ventral striatal activity in the *MLPS* group from day 1 to day 2 was significantly correlated with individual reductions in pain perception, *r* = 0.52, *p* = 0.033, whereas no such correlation emerged in the *FLPS* group, *r* = −0.24, *p* = 0.351 (Fig. 3). This observation is in line with previous reports showing that ventral striatal activation is correlated with various components of pain^44^ and directly links individual pain reduction in our experimental context with activation differences in the dopaminergic system. Importantly, previous studies have implicated activity changes in the dopaminergic system to stress, mediated by a modulation of dopaminergic inputs from the ventral tegmental area by glutamatergic projections from the amygdala^42,45,46^.

Finally, because previous studies suggest that testosterone levels might influence stereotype susceptibility and subsequent behaviour^47^, we further analysed the participants’ saliva testosterone concentration to preclude this confound. The groups did not differ significantly in their testosterone levels (*p* = 0.192).

## Discussion

In the present study, we aimed at investigating the effects of gender-related stereotypes on pain perception and processing. To this end, we employed behavioural pain measures in a high-powered experiment as well as fMRI measures to indicate whether changes in pain report are mirrored by concurrent changes in brain areas associated with pain or pain modulation. We found strong evidence for the influence of stereotypes on pain measures.

Please note that previous studies which have manipulated the participants’ gender-related expectations via instructions to study their effects on pain perception have often used very overt, strong instructions to alter the participants’ expectations^16,17^. This could potentially change the participants’ response patterns by establishing a challenging character to the experimental situation, that is, participants might no longer answer according to their perceived pain, but try to counteract the instruction. Indeed, it seems plausible that such an effect occurred in both studies, with male participants tolerating pain especially long when they were told that females have a higher pain tolerance than men^16^ and participants apparently tolerating pain longer when they were told that the typical man/woman lasts 90 s instead of 30 s in the used pain tolerance task^17^. Given the sensitive nature of this subject, such a confounding challenge effect might even be likely. Another study primed different gender roles before testing the participants’ pain perception by asking participants (males and females) to recall times when they behaved stereotypically masculine or feminine^18^. Again, male participants reported less pain when primed with a feminine gender role, indicating response to a challenge, e.g., “counteracting” these previous instances by behaving more “masculine” during the task. Therefore, we took great care to avoid issuing a challenge to our participants by using very subtle instructions and indeed found no overt challenge effects (see Supplementary Materials). Please note that this lack of challenge might also stem from cultural differences. All previous studies were conducted in the USA, whereas the present study was conducted in Germany. As stereotypes are often culturally different, gender-related stereotypes regarding pain might be more pronounced or considered more relevant in the USA than in Germany, resulting in a greater susceptibility towards feeling challenged by the experimental instructions. Furthermore, previous studies did not include imaging data providing indications of altered pain processing in the brain. We thus believe that, while appreciating the efforts and conclusions of previous studies, the present study presents the first truly conclusive evidence for the influence of gender-specific expectations based on prevalent stereotypes on pain perception and processing.

These findings indicate that pain perception and processing can be affected by a variety of expectations, and these effects are neither limited to treatment expectations that are typically investigated in the context of pain, nor to expectations that are generated by the respective individuals themselves. Rather, stereotypes are characterized as “external” expectations, i.e., expectations other people have about the individual in question^3^. This opens up a new vein of study for the effects of expectations on healthy individuals as well as patients. If expectations differ between experimental groups before, during and/or after interventions, pain reports might not only be confounded by these expectations, but pain processing itself might be affected on a basic level^3,48,49^ and such expectations are potentially based on a multitude of sources. That is, social expectancies might not only indirectly affect a patient by, for example, promoting avoidance behaviour^19^, but might lead directly to changes of that person’s wellbeing.

Which are possible mechanisms underlying the reported effects? A possible explanation lies in differential physiological stress responses to the experimental manipulation between the *MLPS* and *FLPS* group. Indeed, the possible involvement of the dopaminergic system that is indicated by the fMRI data supports this hypothesis, as previous studies have implicated activity changes in the dopaminergic system to stress^42,45,46^, and reported associations of dopaminergic gene variations and individual responses to pain-related stressors^50^. Moreover, stress responses mediate the effects of stereotypes on cognitive abilities (in concert with monitoring processes and thought suppression)^30^. This mediation seems to involve down-regulation of activity in prefrontal circuits which, consequently, impairs working memory processes. Stress responses can also alter pain sensation and reduce pain sensitivity^51–53^. Stress-related physiological processes are further thought to affect large-scale neural network coupling and especially functional brain connectivity between pain-responsive areas in the anterior mid-cingulate cortex and the brainstem^54,55^. These findings provide possible pathways for top-down modulation of pain processing during stress.

In a preliminary attempt to target this question, we measured the cortisol concentration in 54 of our participants on three time points on each experimental day (see Supplementary Results I; Supplementary Fig. S2). Physiological stress responses engage the hypothalamic-pituitary-adrenocortical (HPA) axis which in turn regulates the release of the glucocorticoid cortisol. Typically, a strong trigger is needed to elicit detectable increases in cortisol levels^56^, therefore we included another pain-related stressor at the end of day 2 that has been successful in elevating cortisol levels in previous studies: the Cold Pressor Test (CPT). During this test, participants are asked to hold their right hand in ice-water (0 °C) and to keep it there until they can no longer bear the pain (see Supplementary Methods). After this procedure, participants were asked to rate how painful the test had been perceived. Supplementary Fig. S2 illustrates the effects of the expectancy manipulation on cortisol levels and perceived pain during the CPT. The *FLPS* group reported significantly higher pain ratings than the *MLPS* group. In support of this behavioural effect, the expectancy manipulation groups showed differential physiological stress responses to the CPT with an increase in cortisol levels in the *MLPS* group and no significant change in the *FLPS* group. Interestingly, participants in the *Control* group showed a similar pattern to the *MLPS* group in both, behavioural and physiological data, but differed from the *FLPS* group (for exact results, see Supplementary Results I). Our results thus indicate that participants in the *FLPS* group – in contrast to both other groups – only showed a negligible activation of the physiological stress response, including the release of cortisol, and simultaneously experienced more pain than participants in the other groups. These results imply that stress-induced hypoalgesia might play an important role in the effects of stereotypes on pain processing.

Stress-induced hypoalgesia can rely on opioidergic as well as non-opioidergic neurotransmitter systems^51,53^. Especially the opioidergic system, i.e., the release of endogenous opioids, is a likely candidate for the mediation of stereotype effects on pain because it has been heavily implicated in other expectation effects on pain processing, such as placebo hypoalgesia^21,28,57^. We tested this hypothesis in another, preliminary pharmacological challenge experiment with 31 new participants who were all instructed according to the *MLPS* expectancy manipulation (see Supplementary Methods). Before the expectancy manipulation, however, we either administered the opioid antagonist naloxone or saline. If the hypoalgesic effect observed in our *MLPS* groups depended on the same opioidergic pathway as placebo hypoalgesia, we would expect the effect to be inhibited by naloxone^28,57^. This was not the case, however, neither in pain sensitivity ratings nor in pain threshold measures (see Supplementary Results II; Supplementary Fig. S3). This is a first indication that, surprisingly, stereotype effects on pain perception might be mediated by non-opioidergic transmitter systems.

Please note that as our sample size only consisted of male participants, our results cannot be automatically extrapolated to female participants. Future studies are thus needed to gauge the generalizability of our findings. Moreover, as we did not quantify the participants’ gender role expectations regarding pain before the instructions, we cannot rule out that confirmation and disconfirmation effects systematically vary between the different groups. US questionnaire data suggest that the idea that men are less pain sensitive is generally prevalent^8^, which could lead to a systematic confirmation and systematic disconfirmation effect regarding these stereotypes in the different instruction groups. However, it seems questionable if the prevalence of such stereotypes can be easily translated from one culture (US) to another (Germany). Crucially, our manipulation check questionnaire confirmed that most participants believed our instructions which seems counter-indicative to a strong disconfirmation bias, as participants who strongly believed in one stereotype would be less inclined to believe the instruction.

Taken together, our experiments demonstrate the substantial effects of stereotype-related expectations on pain processing, giving evidence to a causal link between these two instances. Our expectancy manipulations evoked differential behavioural rating patterns and physiological responses on the neural level in response to expectancy-related stimuli. Preliminary results further indicate that a differential physiological stress response might play an integral part in gender-related stereotype modulation of pain, possibly mediated by non-opioidergic neural pathways.

## Materials and Methods

### Participants

We recruited 120 healthy male participants for the main experiment in this study, with 40 participants being randomly assigned to either group (*MLPS*, *FLPS*, *Control*). We focused on male participants being tested by a male experimenter to be able to address our main question – the possibility of an impact of gender-related stereotypes on pain – within a homogenous sample to ensure optimal statistical power.

Sample size was determined by means of power analyses based on previous studies on stereotype effects for cognitive or automatic processes suggesting medium to large effect sizes^2,5^. This lead to a calculation of at least 35 participants per group, assuming an effect size of *d* ≥ 0.60 and a power of 0.80.

A total of 15 participants did not complete data collection due to technical difficulties or were excluded from data analysis because they either did not understand or did not believe our expectancy manipulation, as was assessed in a questionnaire serving as our experimental manipulation check. Of the remaining 105 male participants, 34 received the instruction that men are less pain sensitive than women on the second day (*MLPS* group, mean age 25.85 years ± 0.78 SE_M_), 35 received the instruction that women are less pain sensitive than men (*FLPS* group, 25.54 years ± 0.83), and 36 received no gender-related instruction (*Control* group, 25.53 years ± 0.77). Please note, however, that as pain-related stereotypes are prevalent in the population, they might also have been relevant and effective in the *Control* group, although their effects would be less directed and therefore most likely more diffuse. Because of this, we have treated all results relating to the *Control* group with caution. Exclusion criteria involved neurological and neuropsychiatric diseases, current medication, substance abuse, or skin afflictions on the forearms. The study was approved by the Ethics Committee of the Medical Council of Hamburg and all participants gave written informed consent in accordance with the Declaration of Helsinki.

### Experimental paradigm

**Figure 4:**
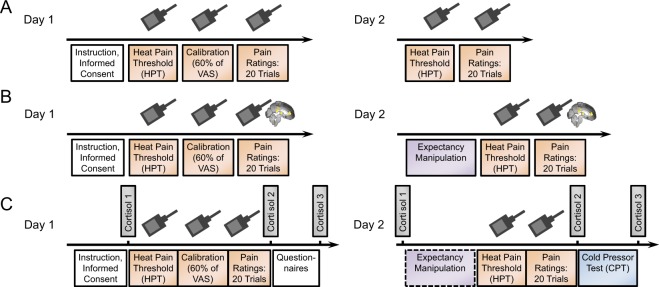
Schemata of the behavioural paradigms employed in the main experiments. (**A**) Behavioural paradigm. After a general instruction on the first day, we measured the participants’ heat pain thresholds, then performed a stimulus calibration to allow for an experimental temperature that elicited pain sensitivity ratings of 60 to 65 on a visual analogue scale (VAS; 0–100) and used that temperature for 20 consecutive pain stimuli. The participants were asked to rate their pain experience after each pain stimulus. On the second day, heat pain threshold measures and pain sensitivity ratings (same temperature as on day 1) were assessed anew. Note that no expectancy manipulation took place here, because only participants in the *Control* group were tested in this basic design. (**B**) fMRI paradigm. Participants were positioned in the MRI scanner prior to the heat pain threshold measurements on both days to assure a similar experimental environment for all pain measures obtained. However, functional imaging measurements were only acquired for the pain sensitivity ratings (20 consecutive pain stimuli). Note that all participants in the fMRI experiment received a gender-related expectancy manipulation at the beginning of the second day, as all participants were either part of the *MLPS* or the *FLPS* group. (**C**) Cortisol paradigm (for physiological results, see Supplementary Results I). At six time points over the two experimental days (three time points per day, T1–T3), saliva samples were taken for subsequent cortisol concentration analysis. Additionally, to elicit stronger cortisol responses, a Cold Pressor Test (CPT) was added at the end of the second experimental day.

All 105 participants completed the behavioural paradigm as shown in Fig. 4, but the basic paradigm was adapted to the needs of the different physiological measures for subsets of these individuals. Seventeen participants completed only the behavioural paradigm (Fig. 4A, all *Control* group), 34 individuals completed the fMRI paradigm (Fig. 4B, n_*MLPS*_ = 17, n_*FLPS*_ = 17), and 54 participants completed the cortisol paradigm (Fig. 4C, n_*MLPS*_ = 17, n_*FLPS*_ = 18, n_*Control*_ = 19).

### Basic Behavioural Paradigm

Participants were measured on two experimental days that were scheduled to be one or two days apart (Fig. 4A). The experimenter was always male and wore a white lab coat. On the first day, participants were told that they would take part in a pain study looking to find individual factors of pain experience and were asked to be as honest as possible in their pain ratings. Heat pain stimuli were applied to the left forearm using a Peltier thermode. We chose heat pain stimuli to increase comparability with previous studies on placebo and nocebo research that also followed similar design protocols and included similar methods^28,32^. We measured the heat pain threshold by slowly increasing stimulus temperature at a rate of 0.3 °C/s, starting at 30 °C. Participants were asked to indicate as soon as they felt the first pain sensation which immediately stopped the temperature increase. This procedure was repeated four times and the four pain threshold temperatures were averaged. We then calibrated the heat pain temperature to elicit a pain rating of about 60 to 65 on a Visual Analogue Scale (VAS) (0–100), ranging from “no pain at all” to “unbearable pain”. In four blocks of three heat pain stimuli of varying temperature each (13 s duration, 10 s plateau), pain sensitivity was assessed by asking the participants to rate these different pain stimuli on the VAS. The temperature falling within the range of 60 to 65 of the VAS was used for the subsequent experiment. After the calibration procedure, participants received 20 heat pain stimuli at the calibration temperature. To assure an individual rating procedure for each stimulus, participants were not explicitly told that the temperature would be constant for all 20 stimuli. The stimulus was preceded by a cue – a red fixation cross on a screen – five seconds before stimulus onset and the red fixation cross remained on the screen until the pain stimulus terminated (13 s duration, 10 s plateau) and the temperature had again dropped to baseline (32 °C). After the stimulus, the VAS rating scale appeared on the screen, ranging from “no pain at all” to “unbearable pain” and participants were asked to indicate their pain experience with a standard computer mouse. The rating procedure and a subsequent inter-trial interval (ITI) during which a black screen was presented lasted for a total of 55 seconds. This break between pain stimuli was implemented to minimize sensitization or habituation effects due to continuous thermal heat stimulation. Altogether each trial lasted 73 seconds and the whole experimental procedure of 20 trials took 24 minutes.

On the second experimental day, the expectancy manipulation for participants in the *MLPS* and *FLPS* groups was applied by subtly briefing those individuals on evolutionary reasons why men and women, respectively, are less sensitive to pain (see *Expectancy Manipulation* below). As part of a questionnaire, they were then asked whether or not they perceived themselves as “masculine” on a 7-point scale ranging from “very feminine” to “very masculine”. They were also asked how important it was for them to be perceived as “masculine” by others on a 7-point scale ranging from “not important at all” to “very important”. As a manipulation check, participants in the *MLPS* group were asked if they believed men to be generally less pain sensitive in standardized tests than women on a 5-point scale ranging from “not true at all” to “absolutely true”. Participants in the *FLPS* group were asked if they believed women to be generally less pain sensitive in standardized tests than men using the same scale. This was intended to screen participants for understanding the instruction and exclude participants possibly feeling challenged by the instruction, as this was a problem in previous studies on the subject^16,17^ (see also Supplementary Methods). Participants who rated the statement as “not true at all” were excluded from further analysis (n = 6). Participants in the *Control* group did not receive further gender-related information. After this procedure, the heat pain threshold was assessed as on the first day and afterwards the heat pain stimuli were applied at the same temperature as on the first day. After the experimental procedure on the second day, participants were briefed on the real objective of the study and were informed about the real relationship between sex, gender, and pain experience that is known so far (for details on the adaptions for the fMRI and Cortisol experiment see Supplementary Methods).

### Expectancy Manipulation

We manipulated the participants’ awareness of common stereotypes regarding their own pain sensitivity by giving participants in the *MLPS* and *FLPS* group an additional information sheet at the beginning of the second day. This information sheet once more reminded the participants of the supposed goal of the study, namely to relate individual factors to pain experience. Participants were also again asked to rate the pain stimuli as honestly as possible. The informational sheet then detailed either that men are generally less sensitive to pain than women, probably due to evolutionary reasons on account of having been hunters prone to physical injury in the past (*MLPS* group) or that women are generally less sensitive to pain than men, probably due to evolutionary reasons on account of having to go through the very painful process of childbirth (*FLPS* group; for the exact wording of the information see Supplementary Methods).

The objective of this gender-related information was to induce the respective expectancy about the participants’ own pain sensitivity, while at the same time avoiding to pose an overt challenge to the participants as such challenges haven been identified to bias the results in previous studies on the subject^16^. As we expected participants who were most strongly concerned with appearing masculine to be most susceptible to perceiving a challenge by the *FLPS* instruction, we tested for a potential challenge by correlating questionnaire scores of the participants’ concern to appear masculine with changes in pain perception due to the instruction, but found no evidence for a challenging character of the instruction (for details see Supplementary Methods; Supplementary Fig. S4).

### Behavioural data analyses

Behavioural data were analysed using SPSS 20. First, mean pain sensitivity ratings of all 20 pain trials were calculated for each participant and each day, separately. Then our main analysis of interest was performed, a 2 × 2 analysis of variance (ANOVA) with the within-subjects factor Time (Day 1 vs. Day 2) and the between-subjects factor Expectancy Manipulation (*MLPS* vs. *FLPS*). We then broadened the ANOVA to also include the *Control* group to allow a better interpretation of the expectancy manipulation effect (Time [Day 1 vs. Day 2] × Expectancy Manipulation [MLPS vs. FLPS vs. Control]). Subsequent simple effects ANOVAs looked at the differences between the *MLPS* and *Control* group and the *FLPS* and *Control* group, respectively (Time [Day 1 vs. Day 2] × Expectancy Manipulation [MLPS × Control] and Time [Day 1 vs. Day 2] × Expectancy Manipulation [FLPS × Control]).

Mean pain threshold temperatures were calculated for each participant and day, separately. Again, our main analysis of interest was a two-way ANOVA with the within-subjects factor Time (Day 1 vs. Day 2) and the between-subjects factor Expectancy Manipulation (*MLPS* vs. *FLPS*). The following analyses were computed as described for the pain sensitivity ratings. For technical reasons, one participant of the *Control* group had to be excluded from the pain threshold analysis.

Cold Pressor Test (CPT) pain ratings and pain duration were analysed using separate one-way ANOVAs with the between-subjects factor Expectancy Manipulation (*MLPS* vs. *FLPS* vs. *Control*) and independent-samples *t* tests for pairwise comparisons between groups. For technical reasons, one participant of the *Control* group had to be excluded from the CPT pain rating analysis.

Note that our results figures employ different error bars dependent on the underlying statistical model of the calculation to optimize the interpretational value of the graphs. The type of the respective error bar is denoted at the y-axis of each figure (SE_M_ = standard error of the mean; SE_D_ = standard error of the between-subjects difference between two means^58^; SE_PD_ = standard error of the (within-subjects) paired difference between two means^58^; SE_LM_ = Loftus-Masson within-subjects standard error for repeated-measures ANOVA^59^).

### fMRI parameters and data analyses

#### fMRI parameters

Imaging data were obtained on a 3 Tesla system, equipped with a 32-channel head coil. A T2*-weighted standard gradient echo planar imaging sequence was used to measure BOLD responses (repetition time 2.58 s; echo time 26 ms; flip angle 80°; field of view 220 × 220 mm^2^; GRAPPA PAT Factor 2). Each volume contained 42 transversal slices (voxel size 2 × 2 × 2 mm^3^; 1 mm gap). Volumes were individually tilted by approximately 30° relative to the AC-PC plane to allow whole-brain acquisitions including the brainstem. The first 4 volumes of each session were discarded to account for T1 saturation effects. High resolution T1 scans were acquired using an MPRAGE sequence with a voxel size of 1 × 1 × 1 mm^3^.

#### fMRI data analysis

fMRI data were preprocessed and statistically analysed by using SPM12 (Wellcome Department of Imaging Neuroscience, London, UK) implemented in Matlab R2014a. Data preprocessing consisted of motion correction (realignment), coregistration of the individual anatomical T1 image to the functional images, spatial normalization to MNI space using DARTEL based on segmented T1 scans. The DARTEL estimation used templates provided by the VBM 8 toolbox (http://dbm.neuro.uni-jena.de/vbm). All fMR images were smoothed using a 6 mm (FWHM) isotropic Gaussian kernel. We used a high-pass filter to cut off all slow signal drifts with periods longer than 128 seconds and a correction for temporal autocorrelations was performed using a first-order autoregressive model.

fMRI data analysis was based on the general linear model approach as implemented in SPM. For each individual, the design matrix consisted of 10 regressors for each session. Each regressor modelled the activation in a time bin (one TR, i.e., 2.58 s) after stimulus onset, where time point zero was defined as the first appearance of the red fixation cross (cue). The entire set of regressors thus covered a time period of 25.8 seconds after cue presentation. This finite impulse response (FIR) model has the advantage that no *a priori* assumptions about hemodynamic response patterns have to be made, and at the same time it can test for specific activation patterns at every time period. We focused our analyses on the anticipation phase (i.e., the second bin spanning 2.58s-5.16 s after cue onset) and the early (10.32 s–15.48 s) and late (15.48 s–23.22 s) pain period. The rigid body transform motion parameters from the realignment stage were included as additional regressors. After model estimation at the first-level comparing parameter estimates between day 1 and day 2 for the anticipation, early pain and late pain phase, the resulting contrast images were used for second-level group analyses. At the second level, a two sample *t*-test was employed comparing the changes from day 1 to day 2 between the *MLPS* and the *FLPS* group.

For all imaging data analyses, results were corrected for multiple comparisons within anatomically defined regions of interest i.e. all voxels of all regions of interest (ROIs). All *p* values are reported with the corrected value as well as the uncorrected value to increase transparency. ROIs were based on anatomical masks taken from the Harvard-Oxford atlas (normalized to the DARTEL templates as provided by the VBM 8 toolbox). ROIs comprised of bilateral anterior cingulate cortex (ACC), the insula including the parietal and frontal operculum, the basal ganglia (ventral striatum), the thalamus, and the amygdala (see Supplementary Fig. S5 for ROI locations overlaid on the mean T1 image from all participants). The significance threshold for corrected *p* values was defined as *p*_corr_ < 0.05. For illustration purposes only, statistical maps were thresholded at *p* < 0.005, uncorrected, with a voxel extent of minimum 10 and overlaid on the mean structural image of all subjects. All activations are reported using x, y, z coordinates based on the used template, which is in Montreal Neurological Institute (MNI) standard space.

### Testosterone analysis

Saliva samples were obtained (see Supplementary Methods for details) and frozen at −20 °C until study completion. In preparation for hormone analysis, the samples were thawed and centrifuged at RCF 604 x g for five minutes (i.e., 3000 rpm in a centrifuge) to separate them from mucin and other residuals. The five morning samples were combined to an aliquot by extracting 2 ml of clear, colourless supernatant from each of the five Eppendorf tubes. Samples containing traces of blood were excluded. A Testosterone Luminescence Immunoassay was used to determine testosterone concentrations in the aliquot. The sensitivity of the Testosterone Luminescence Immunoassay is denoted as 1.8 pg/mL.

## Supplementary information

Supplementary Information

Dataset 1

## Data Availability

All data (except for fMRI data) are available with the paper and can be found in the Supplementary Information Data file.
