# Supplementary material for: How Stereotypes Affect Pain
Source: Sci Rep. 2019 Jun 13;9:8626. doi: 10.1038/s41598-019-45044-y (PMC6565709; doi:10.1038/s41598-019-45044-y)
Supplement: Supplementary file 1 — Supplementary Information [file 41598_2019_45044_MOESM1_ESM.pdf]

How Stereotypes Affect Pain

Supplementary Information

Katharina A. Schwarz[1,2]*, Christian Sprenger[1], Pablo Hidalgo[1], Roland Pfister[2], Esther K. Diekhof[3], and Christian Büchel[1]

[1]Department of Systems Neuroscience, University Medical Center Hamburg-Eppendorf, Hamburg, Germany.

[2]Institute of Psychology, University of Würzburg, Würzburg, Germany.

[3]Institute of Human Biology, University of Hamburg, Hamburg, Germany.

*Corresponding Author:

Dr. Katharina A. Schwarz

Röntgenring 11

97070 Würzburg

Germany

+49-931-31-88655

katharina.schwarz@uni-wuerzburg.de

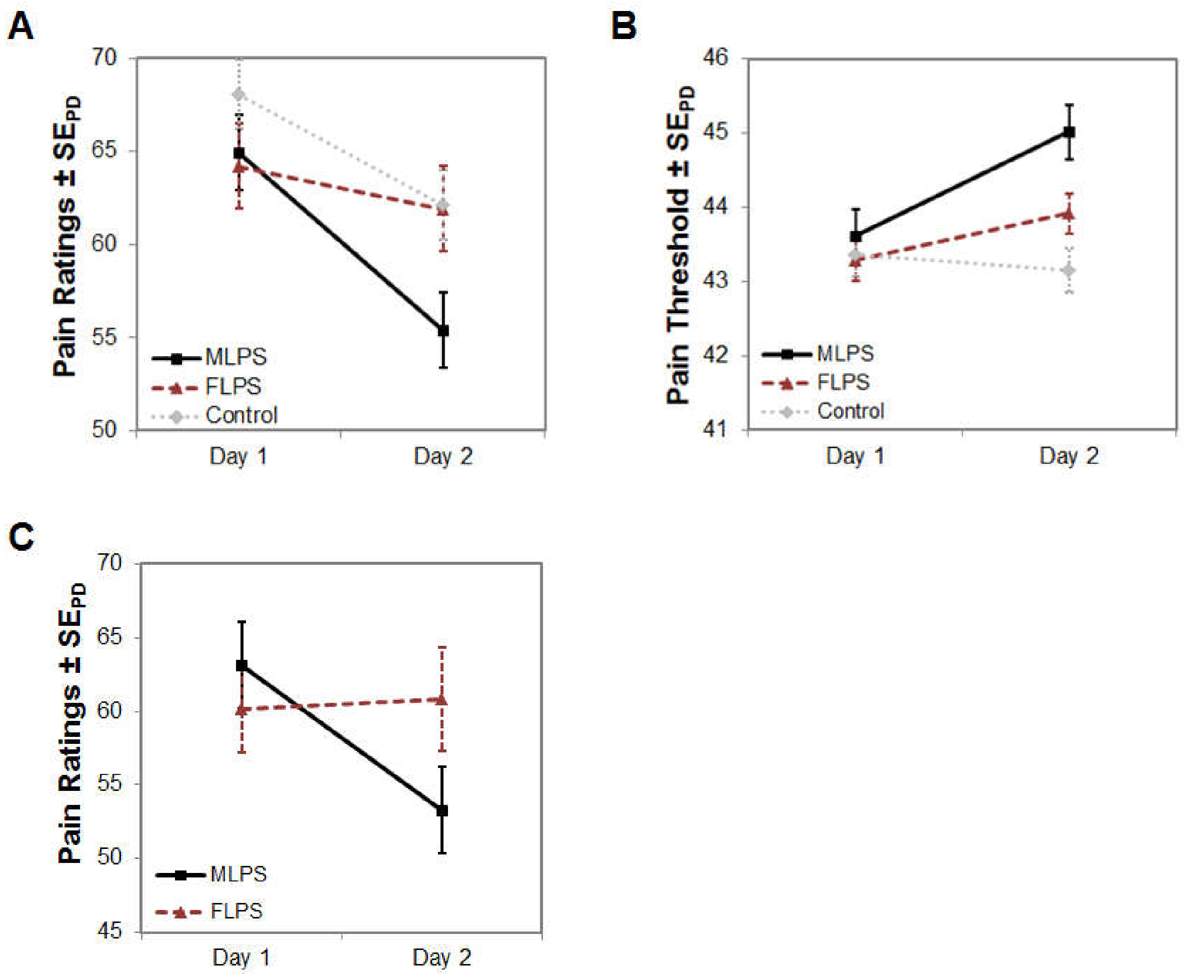

Supplementary Figure S1. Raw data of pain sensitivity ratings. A. Pain sensitivity ratings for each group and each day. The effect of gender-related expectancy manipulation is significant both, when comparing the two main groups of interest (interaction *Time* x *Expectancy Manipulation*; *MLPS* vs. *FLPS*) as well as in the omnibus analysis (interaction *Time* x *Expectancy Manipulation*; *MLPS* vs. *FLPS* vs. *Control*). Moreover, the pain ratings on the first day were generally higher than on the second day, $F(1,102) = 24.97$, $p < .001$, $\eta_p^2 = .20$, however group-wise paired t-tests reveal only significant differences between day 1 and day 2 in the *MLPS,* $t(33) = 4.75$, $p < .001$, and *Control*, $t(35) = 3.17$, $p = .003$, groups, not in the *FLPS* group, $t(34) = 1.01$, $p = .319$. The groups did not differ significantly on day 1, $F < 1$. Scale: 0-“no pain at all”, 100-“unbearable pain”. Please note that the *Control* group primarily differed from the expectancy manipulation groups in that their expectancy was not altered from what the participants

already believed. Previous literature suggests that the *MLPS* expectancy manipulation likely reflects common stereotypes, possibly accounting for some of the similarities in result patterns between the *MLPS* and the *Control* group [8]. However, this explanation has to be treated with caution as stereotypical beliefs prevalent, e.g., in US society, might not be easily transferable to German society. **B.** Pain threshold temperatures in °C for each group and each day. The interaction *Time* x *Expectancy Manipulation* is only marginally significant when comparing only the *MLPS* and the *FLPS* group, $F(1,67) = 2.83$, $p = .097$, $\eta_p^2 = .04$, but all other interactions (overall and simple effects) show significant differences: the overall interaction including the *Control* group is highly significant, $F(2,101) = 6.51$, $p = .002$, $\eta_p^2 = .11$) and the simple effects ANOVAs reveal significant differences especially between the *MLPS* and *Control* group, $F(1,67) = 11.55$, $p = .001$, $\eta_p^2 = .15$, but also between the *FLPS* and *Control* group, $F(1,68) = 4.36$, $p = .041$, $\eta_p^2 = .06$. The general response pattern clearly indicates that the *MLPS* group shows the strongest increase in pain threshold temperature on day 2 compared to day 1. There is again a strong effect of *Time*, $F(1,101) = 11.39$, $p = .001$, $\eta_p^2 = .10$, with a general increase in pain threshold temperature on the second day compared with the first one over all participants. Paired *t*-tests calculated for each group show again that this effect is strongest for the *MLPS* group, $t(33) = -3.79$, $p = .001$. The day 1 vs. day 2 comparison is also significant for the *FLPS* group, $t(34) = -2.35$, $p = .025$), but not for the *Control* group ($|t| < 1$). **C.** Raw pain sensitivity ratings for the fMRI experiment; scale: 0-“no pain at all”, 100-“unbearable pain”. The critical interaction *Time x Expectancy Manipulation* was significant, $F(1,32) = 5.28$, $p = .028$, $\eta_p^2 = .14$, and the main effect of *Time* showed a non-significant trend, $F(1,32) = 3.91$, $p = .057$, $\eta_p^2 = .11$. The main effect of *Expectancy Manipulation* did not approach significance, $F < 1$. Paired *t*-tests calculated for each group showed a pronounced difference between day 1 and day 2 for the *MLPS* group, $t(33) = -3.34$, $p = .004$, but not for the *FLPS* group, $t(33) = 0.21$, $p = .837$.

Supplementary Results I

Cortisol Results

We measured cortisol concentration in 54 participants on three time points on each experimental day (*Figure 4C*; n_{MLPS} = 17, n_{FLPS} = 18, $n_{Control}$ = 19). Physiological stress responses engage the hypothalamic-pituitary-adrenocortical (HPA) axis which in turn regulates the release of the glucocorticoid cortisol. Typically, a strong trigger is needed to elicit detectable increases in cortisol levels [56], therefore we included another pain-related stressor at the end of day 2 that has been successful in elevating cortisol levels in previous studies: the Cold Pressor Test (CPT). During this test, participants are asked to hold their right hand in ice-water (0°C) and to keep it there until they can no longer bear the pain (see *Supplementary Methods* below for further details). After this procedure, participants were asked to rate how painful the test had been. *Supplementary Figure S2* illustrates the effects of the expectancy manipulation on cortisol levels and perceived pain during the CPT. Our main analysis of interest again concerned the difference between the expectancy manipulation groups, i.e., *MLPS* vs. *FLPS*. The *FLPS* group reported significantly higher pain ratings than the *MLPS* group, $t(33) = -2.13$, $p = .041$, $d = -0.72$. In support of this behavioural effect, the expectancy manipulation groups showed differential physiological stress responses to the CPT with an increase in cortisol levels in the *MLPS* group and no significant change in the *FLPS* group, interaction *Time* x *Measurement* x *Expectancy Manipulation*: $F(2,64) = 3.31$, $p = .043$, $\eta_p^2 = .09$.

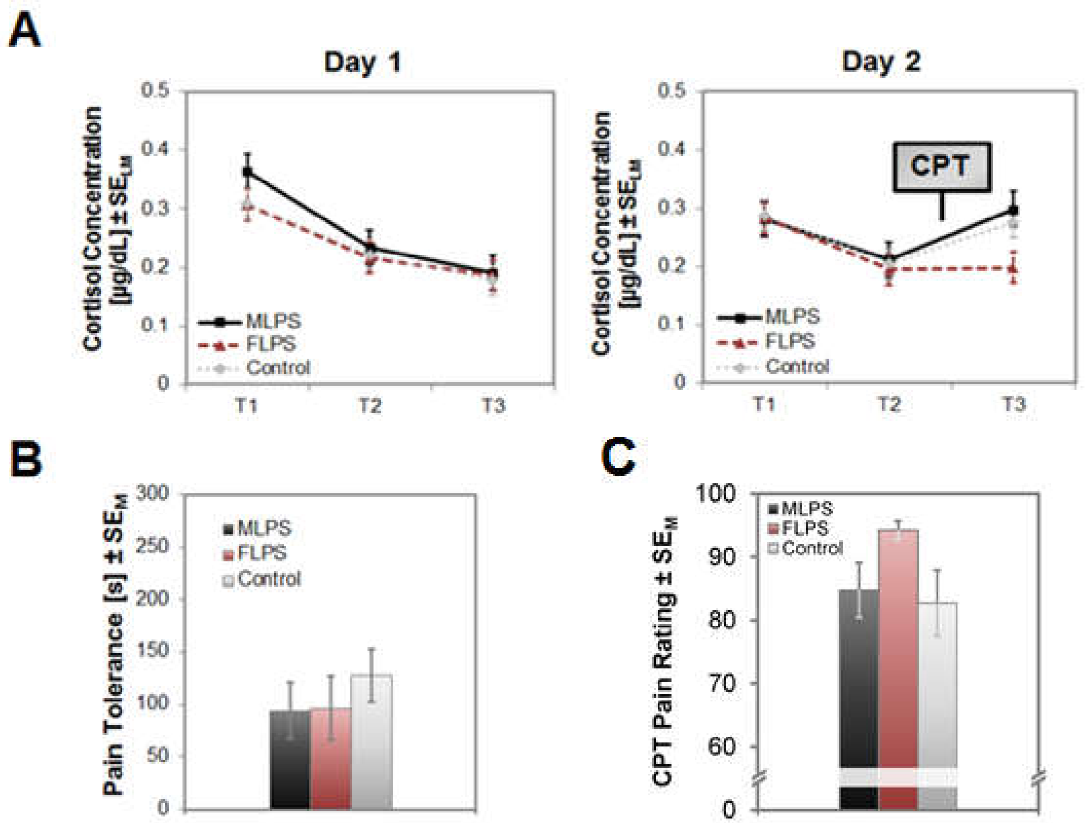

Supplementary Figure S2. Results of the Cortisol experiment. A. Cortisol concentrations for each group and time point (T1-T3) on each experimental day. **B.** Pain tolerance as measured by the Cold Pressor Test (CPT). There were no significant differences between any of the groups ($F < 1$); the effects observed in the rating data are therefore not confounded by differential exposure to the cold pain stimulus. **C.** Cold Pressor Test (CPT) pain ratings for each group (scale: 0-“no pain at all”, 100-“unbearable pain”).

To better interpret this effect, we also investigated a *Control* group. Notably, the cortisol concentration of the *Control* group did not lie symmetrically between the *FLPS* and *MLPS* group as is suggested by a non-significant linear contrast (*MLPS* > *Control* >

FLPS), $F(1,50) = 2.09$, $p = .149$, but was much closer to the concentration change of the *MLPS* group. When comparing the raw cortisol concentration of the last measurement on each day, the *FLPS* group only showed a weak and non-significant stressor-related increase ($|t| < 1$), whereas the *MLPS* and the *Control* group responded with a significant increase in cortisol levels on day 2 as compared to day 1, (ps < .033, one-tailed). The *MLPS* and *Control* group also showed similar rating patterns ($|t| < 1$) whereas the *FLPS* and *Control* group differed significantly, $t(34) = 2.11$, $p = .043$, $d = 0.71$, with the *Control* group reporting less perceived pain than the *FLPS* group during the CPT. A possibility to explain this asymmetry lies with a "default" stereotype which holds that males are less pain sensitive which is accordance with US questionnaire data [8]. In this light, it is not surprising to see a similar pattern in the *Control* and *MLPS* group. However, it seems questionable if such stereotypes can be easily translated from one culture (US) to another (Germany).

Supplementary Results II

Naloxone Results

The release of endogenous opioids is a well-documented mechanism of placebo hypoalgesia [21, 28], during which the descending opioidergic pain pathway is activated, leading to inhibition of nociceptive processing at the spinal level. This process and subsequent behavioural hypoalgesic effects can be inhibited by the administration of naloxone, an opioid antagonist [28]. Thus, we would expect the hypoalgesic effect observed in our *MLPS* groups to be inhibited by naloxone, if the effect were to depend on the same descending opioidergic pathway.

To investigate this hypothesis, we additionally tested 40 healthy male participants for the naloxone experiment (these participants are not included in the original count of 120 participants). Nine individuals did not complete data collection due to technical difficulties or were excluded later because they felt uncomfortable for longer than 15 minutes after the intravenous line was inserted. Of the remaining 31 participants, 15 received saline and 16 received the opioid antagonist naloxone. All participants were given the same stereotype expectation manipulation and were instructed that men are less pain sensitive than women (*MLPS* expectancy manipulation; for more details see *Supplementary Methods* below).

Our results did not support the hypothesis that stereotype effects in pain are mediated by opioidergic pathways. In this experiment, the naloxone group did not differ from the saline group in the decrease of pain sensitivity on the second day compared to the first day, neither in pain sensitivity ratings, interaction *Time* x *Opioid State*: $F(1,29) = 1.03$, $p = .318$, nor in pain threshold measures, interaction *Time* x *Opioid State*: $F(1,29) <$

1 (*Supplementary Figure S3*). Both groups showed the previously described reduction in pain sensitivity from the first day to the second with an 18.2% decrease for the saline group and a 13.6% decrease for the naloxone group. While there is a descriptively steeper reduction in pain sensitivity in the saline group than in the naloxone group, this change is unlikely to underlie the observed behavioural effects alone, especially regarding the pain threshold measures. We therefore have no evidence suggesting that an opioidergic mechanism is at play in the effects of stereotypes on pain processing though this null effect should be interpreted with caution given the limited sample sizes of this between-group comparison.

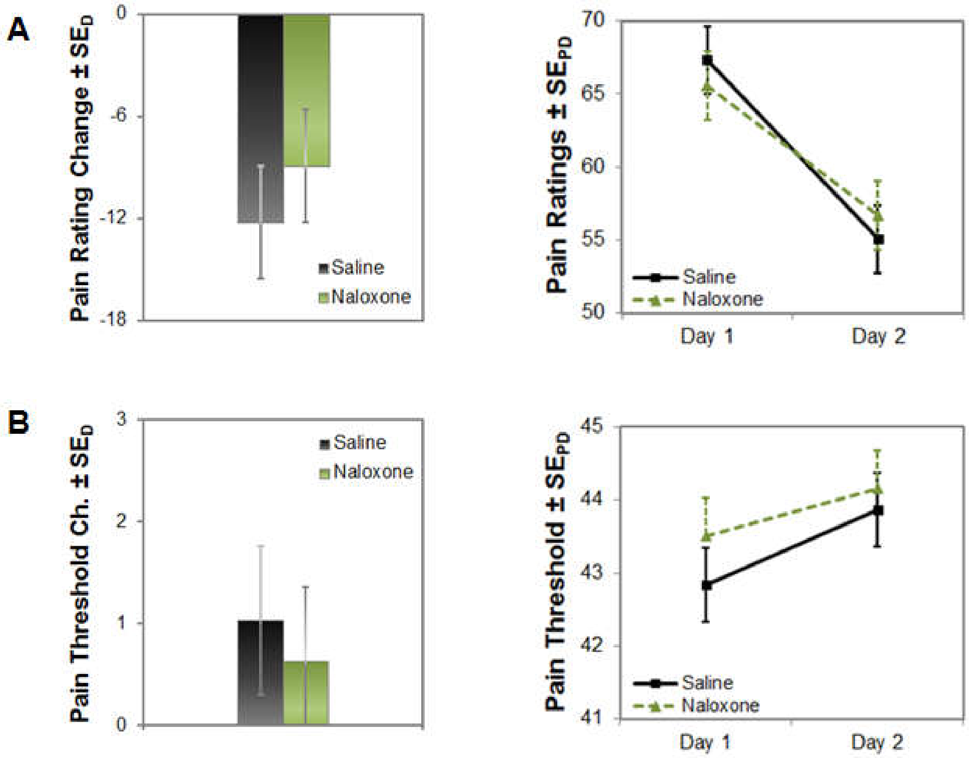

Supplementary Figure S3. Results of the naloxone experiment. A. Changes in pain sensitivity ratings (day 2 – day 1; left panel) and corresponding raw scores (right panel) of the naloxone experiment. The expectancy manipulation was the same in both groups

("men are less pain sensitive") and pain ratings were generally higher on the first day than on the second day, $F(1,29) = 41.60$, $p < .001$, $\eta_p^2 = .59$. Subsequent paired *t*-tests for each group indicated the difference between day 1 and day 2 to be significant for both, the *Saline* and the *Naloxone* group, *Saline*: $t(14) = 5.30$, $p < .001$, *Naloxone*: $t(15) = 3.84$, $p = .002$. Even though the difference between day 1 and day 2 was descriptively smaller for the *Naloxone* group (as would be expected when assuming the effect to be mediated by the release of endogenous opioids), the corresponding interaction *Time* x *Opioid State* did not reach significance, $F(1,29) = 1.03$, $p = .318$. The opioid antagonist naloxone therefore does not seem to inhibit the hypoalgesia effect observed in the *MLPS* group in our experiments (*Figure 1*), even though the corresponding descriptive trend seems to be in accordance with recent findings on Naloxone effects in rodents [53]. **B.** Changes in pain threshold temperature (day 2 – day 1; left panel) and corresponding raw scores (right panel). Again, there was no significant interaction *Time* x *Opioid State,* $F < 1$, suggesting that naloxone alone did not inhibit the rise in pain threshold temperature on the second day in the *MLPS* groups in our main experiment (*Supplementary Figure S1*). Pain threshold temperatures on the first day were generally lower than on the second day, $F(1,29) = 5.50$, $P = .026$, $\eta_p^2 = .16$), although group-wise paired *t*-tests showed that the difference between day 1 and day 2 was only marginally significant in the *Saline* group, $t(14) = -2.06$, $p = .059$, and not significant in the *Naloxone* group, $t(15) = -1.27$, $p = .224$).

Supplementary Methods

Expectancy Manipulation

We manipulated the participants' awareness of common stereotypes regarding their own pain sensitivity by giving participants in the *MLPS* and *FLPS* group an additional information sheet at the beginning of the second day. This information sheet once more reminded the participants of the supposed goal of the study, namely to relate individual factors to pain experience. Participants were also again asked to rate the pain stimuli as honestly as possible.

Within this information sheet, the following phrases were included for the *MLPS* group (translated from German): "We know by now that various personal factors have a strong and predictable influence on pain perception. For example, pain research shows consistently that men react less strongly to pain in standardized tests than women do, i.e., they seem to be less pain sensitive than women. From an evolutionary point of view, a development of such differences is easy to explain, in this example it is argued that the high risk of painful injuries during hunting or while defending resources which are all actions primarily performed by our male ancestors might be responsible. To improve agility with small injuries, the pain perception in men was probably desensitized over the course of human evolution. Further differences include, e.g., personality traits, age, and hormone levels. (…)"

The information sheet for the *FLPS* group contained the following phrases (translated from German): "We know by now that various personal factors have a strong and predictable influence on pain perception. For example, pain research shows consistently that women react less strongly to pain in standardized tests than men do,

i.e., they seem to be less pain sensitive than men. From an evolutionary point of view, a development of such differences is easy to explain, in this example it is argued that the very painful and highly relevant parturition for which the female body is already prepared might be responsible. Further differences include, e.g., personality traits, age, and hormone levels. (…)"

The objective of this gender-related information was to induce the respective expectancy about the participants' own pain sensitivity, while at the same time avoiding to pose an overt challenge to the participants. We suspected that if such a challenge were issued, especially participants in the *FLPS* group would be driven to refute the notion that they might be seen as "inferior" to women regarding pain sensitivity and thus not responding honestly but according to their own agenda. To assure that this latter objective was met, we measured the self-perceived masculinity of the participants by asking them whether or not they perceived themselves as "masculine" and how important it was for them to be perceived as "masculine" by others. The summed answers of these two questions ranging from 2 ("very feminine" and "not important at all" to 14 "very masculine" and "very important") were correlated with the participants' rating difference (day 1 – day 2). If a challenge was issued to the participants, we expected a strong positive correlation of the pain rating difference with the self-perceived masculinity score, i.e., the higher the self-perceived masculinity the lower the rating on the second day compared with the first day should be. *Supplementary Figure S4* shows the respective correlations. No correlation for either stereotype group approached significance ($p > .482$), and as can easily be seen, the *FLPS* group descriptively even showed a negative correlation of the

pain rating difference with the self-perceived masculinity score, if anything. We interpret these findings as indication that indeed no challenge was perceived by the participants.

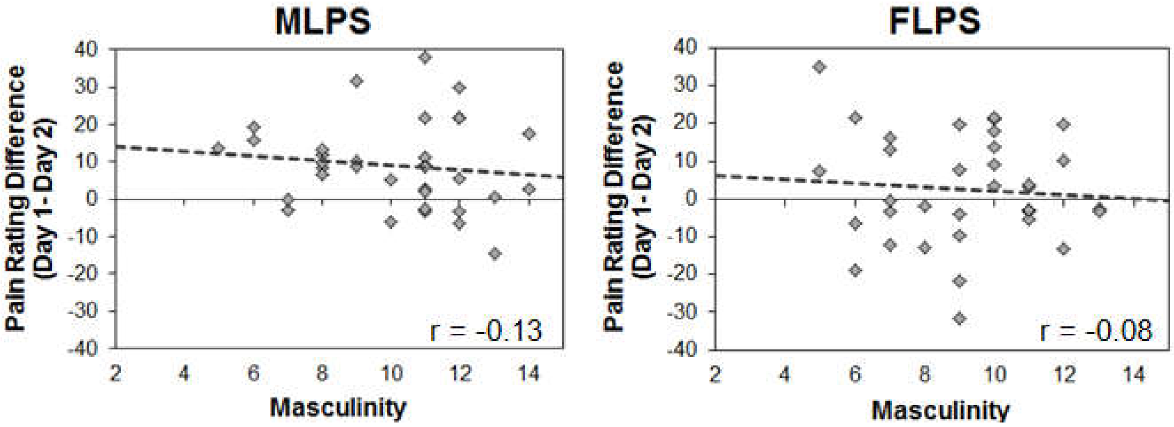

Supplementary Figure S4. Masculinity and pain rating changes. Correlation of the pain rating difference (day 1 – day 2) with the self-perceived masculinity score for either stereotype group. No correlation approached significance and especially the correlation pattern in the *FLPS* group indicates that no challenge was issued due to our expectancy manipulation.

fMRI Paradigm

The experimental design of the fMRI experiment showed slight adaptations to the basic behavioural paradigm (*Figure 4B*). The instructions remained the same, but all pain measurements were obtained while the participants were lying in the MR scanner on both days. No imaging data were acquired during pain threshold measures and during stimulus calibration; however, functional imaging data were obtained during the subsequent pain

rating procedure on both days. On the first day, the measurements were concluded with high resolution anatomical T1 scans (see *fMRI parameters*).

The pain rating procedure was slightly altered to adapt the experimental design to the requirements of the changed location and later fMRI data analysis. The pain stimulus was again preceded by a red fixation cross on a screen five seconds before stimulus onset, and the red fixation cross (cue) remained on the screen for the duration of the pain stimulus (13s duration, 10s plateau). The pain stimulus was followed by a short jitter period during which the screen turned black, lasting for a randomized time between 2 and 5 seconds with an average of 3.5 seconds. After the jitter, the VAS rating scale appeared on the screen, ranging from "no pain at all" to "unbearable pain" and participants were asked to indicate their pain experience via button presses. The cursor on the rating scale appeared at a random place on the scale and while the left or right button was pressed, it moved continuously along the scale until the button was released. When participants were satisfied with their rating, they confirmed their choice by pressing a third button. Participants were asked to complete their rating within 15 seconds. After they confirmed their rating choice or after the 15 seconds had passed, the screen showed a white fixation cross for 30 seconds, and participants were asked to fixate the cross to reduce head movements. The whole pain rating procedure took 22 minutes.

As an additional measure, we asked our participants in the fMRI experiments to give saliva samples on the morning of the second experimental day for testosterone analysis. Participants were equipped with 2 ml polypropylene Eppendorf tubes and an extensive informational sheet detailing the saliva sample procedure. Saliva collection started in the morning after waking up and before breakfast. All fMRI participants collected

five saliva samples in Eppendorf tubes with a time gap of 30 minutes in between, yielding a total sampling time of 2 hours. This procedure allowed to control for the episodic secretion pattern of steroid hormones and gave a representative sample of the participants' current hormone levels. Participants were instructed not to eat, smoke or drink anything but water during the 2 hours sampling time and they were asked to restrict themselves to a vegan diet for 12 hours before sampling onset. Three participants had to be excluded from this analysis because they failed to comply with the saliva sampling instruction.

Cortisol Paradigm

The basic behavioural design was adapted to the needs of the cortisol measurements in the Cortisol experiment (*Figure 4C*). One or two days before the start of the experiment, participants were asked to come to the laboratory and were informed about the saliva sampling procedure including diet restrictions (vegan diet for 12 hours before their appointment on the first experimental day, no food or drink but water for two hours before the start of the experiment, no smoking for two hours before the start of the experiment). They were asked to follow a regular sleep-wake cycle over the course of the experiment, i.e., to go to sleep at similar times at night, and to refrain from alcohol use and extensive activity right before sleeping. These instructions were intended to assure that different sleeping and waking patterns did not interfere with comparable cortisol release on both experimental days. We also provided the participants with ActiWatches which they were instructed to wear at all times, to enforce compliance with our sleeping instructions. Participants were also asked to wake up at least three hours before the start

of the experiment. Note that all experimental procedures took place in the afternoon to avoid the strong cortisol fluctuations in the first hours after waking up.

On the first experimental day, participants received the same general instruction as described above and gave their consent to the experiment. We then asked them to collect their saliva in two 2 ml polypropylene Eppendorf tubes (first sample). The experimental procedure progressed as described in the main methods section with pain threshold measures, stimulus calibration, and the pain intensity rating procedure. After the pain stimulation, we waited for 10 minutes and then asked our participants to again collect two saliva samples (second sample). The participants were instructed to fill out questionnaires after this sampling and at the end of the first experimental day collected two more saliva samples (third sample). On the second experimental day, participants collected two saliva samples before the testing phase (fourth sample), and then received the expectancy manipulation in case of the *MLPS* and *FLPS* groups, or no further information in case of the *Control* group. The subsequent pain stimulation followed the experimental design as described in the main methods. After the testing phase, we waited for 10 minutes and then asked our participants to collect two saliva samples (fifth sample). Because cortisol level increase is primarily detectable after exposure to a strong stressor [32], the sampling was followed by a CPT. This test was surprising for the participants as they were informed about it only right before it took place. During the CPT, participants immersed their right hands into a bucket of ice-water (0°C). They were instructed to keep their hands in the water until they could not bear the pain anymore. The duration of their stay in the water served as a pain tolerance measure that was recorded by the experimenter with a stop watch. The participants were free to remove their hands and

terminate the test at any time, but they were asked to be as honest as they possibly could be about their pain tolerance and not end the test prematurely. If they had not removed their hands after 10 minutes, they were asked to do so, but the participants did not know about this limitation before the test. After the CPT, the participants answered the question "how painful was the test for you on average?" on a scale ranging from "no pain at all" to "unbearable pain" presented to them on a computer screen. We waited for another ten minutes until the participants were asked to give two more saliva samples (sixth sample).

Cortisol Analysis

Saliva samples were obtained as described above. The post-stressor 10 minutes waiting period was chosen to optimize cortisol increase detection [55]. The samples were then frozen at -20°C until study completion. In preparation for hormone analysis, the samples were thawed and centrifuged at RCF 604 x g for five minutes (i.e., 3000 rpm in a centrifuge) to separate them from mucin and other residuals. The two saliva samples per time point were combined to an aliquot by extracting 5ml of clear, colourless supernatant from each of the two Eppendorf tubes, resulting in 6 aliquot samples per participant. Samples containing traces of blood were excluded. A Cortisol Luminescence Immunoassay was used to determine cortisol concentrations in the aliquot. The sensitivity of the Cortisol Luminescence Immunoassay is denoted as 0.005 µg/dL. For technical reasons, one participant of the *FLPS* group had to be excluded from the cortisol concentration analysis. Cortisol concentrations were analysed by a 2 x 3 x 2 ANOVA with the within-subjects factors *Time* (Day 1 vs. Day 2) and *Measurement* (T1 vs. T2 vs. T3) and the between-subjects factor *Expectancy Manipulation* (*MLPS* vs. *FLPS*).

We opted for measuring salivary cortisol rather than blood cortisol for several reasons. First, the repeated collection of blood samples might pose a pain stressor in itself, which would pose a significant confound. Second, the concentration of free bioactive cortisol is considered a reliable indicator of environmental perturbations, i.e., stressors [S1]. Cortisol determined from saliva represents only the free bioactive fraction of cortisol in the system, which in contrast to the bound fraction of cortisol, can pass the membrane of the salivary glands. In contrast, plasma and serum cortisol contain both the free and bound fraction of cortisol (i.e., total cortisol). Following the stressor and the rise of blood cortisol, which takes between 10 and 30 minutes, the transfer from blood to saliva takes place rather rapidly (within 2-3 minutes), thus providing a prompt measure of the stress response. Finally, the analysis of free cortisol in saliva offers a convenient and reliable test with equivalent performance as the analysis of bioactive cortisol from human blood. The luminescence-enhanced enzyme immunoassay we used shows an excellent analytical and functional sensitivity for the routine determination of cortisol from human saliva [S2].

Naloxone Experiment

Participants

We additionally tested 40 healthy male participants for the naloxone experiment (these participants are not included in the original count of 120 participants). Nine individuals did not complete data collection due to technical difficulties or were excluded later because they felt uncomfortable for longer than 15 minutes after the intravenous line was inserted. Of the remaining 31 participants, 15 received saline (mean age 25.13 years

± 0.69 SE_M) and 16 received the opioid antagonist naloxone (24.06 years ± 0.84). All participants were given the same stereotype expectation manipulation and were instructed that men are less pain sensitive than women (*MLPS* expectancy manipulation). Exclusion criteria involved neurological diseases, cardiovascular diseases, current medication, substance abuse, illegal drug consumption in the last 4 weeks before the first day of the experiment, or skin afflictions on the forearms. All participants gave written consent and the consent form included information about the experimental procedures, the thermal stimulation, and about the possible adverse effects of naloxone. Participants were not informed about the actual purpose of the study until debriefing at the end of the second experimental day. The study was approved by the Ethics Committee of the Medical Council of Hamburg and all participants gave written consent in accordance with the Declaration of Helsinki.

Naloxone Paradigm

The experimental design in the naloxone experiment shows slight adaptations to the basic behavioural paradigm described in the *Methods* section. After giving their informed consent on the first day, participants were tested for current drug use (including THC and opiates) using commercially available urine tests. A standard resting electrocardiography was performed to assure that no unknown cardiac arrhythmia existed. Participants then followed the experimental paradigm as detailed in the main methods. On the second experimental day, participants received either the drug injection or saline solution about 15 minutes before the start of the testing phase (see *Drug Administration* below). After the 15 minutes period, the expectancy manipulation was

performed and the subsequent testing period precisely followed the basic behavioural experimental design as described above.

Drug Administration

About 15 minutes before the start of the testing phase, we administered a bolus dose of 0.15 mg/kg naloxone or the same amount of saline via an intravenous line inserted in the right forearm in a double-blind study design. We also administered an additional intravenous infusion dose of 0.2 mg/kg/h naloxone or saline, shortly after bolus administration. This dosing regimen leads to stable naloxone plasma concentrations which correlate strongly with the concentration in the central nervous system.

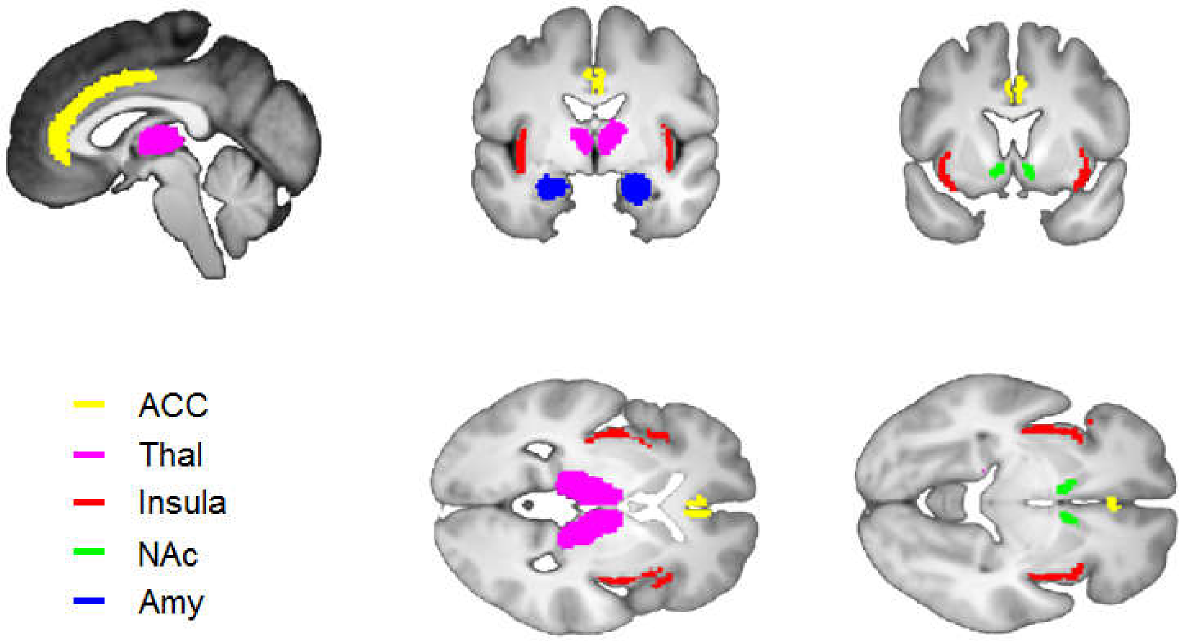

Supplementary Figure S5. ROI locations for fMRI analysis. ROI locations for fMRI analysis, overlaid on the mean T1 image from all participants. Coordinates were taken from the Harvard-Oxford atlas, normalized to the DARTEL templates as provided by the VBM 8 toolbox.

Supplementary References

S1. Inder, W. J., Dimeski, G. & Russell, A. Measurement of salivary cortisol in 2012 - laboratory techniques and clinical indications. *Clin. Endocrinol.* **77**(5), 645-651 (2012).

S2. Westermann, J., Demir, A. & Herbst, V. Determination of cortisol in saliva and serum by a luminescence-enhanced enzyme immunoassay. *Clin. Lab*. **50**(1-2), 11-24 (2004).
