# Supplementary material for: How Stereotypes Affect Pain
Source: Sci Rep. 2019 Jun 13;9:8626. doi: 10.1038/s41598-019-45044-y (PMC6565709; doi:10.1038/s41598-019-45044-y)
Supplement: Supplementary file 2 — Dataset 1 [file 41598_2019_45044_MOESM2_ESM.pdf]

How Stereotypes Affect Pain

Supplementary Information: Data

Katharina A. Schwarz[1,2]*, Christian Sprenger[1], Pablo Hidalgo[1], Roland Pfister[2], Esther K. Diekhof[3], and Christian Büchel[1]

[1]Department of Systems Neuroscience, University Medical Center Hamburg-Eppendorf, Hamburg, Germany.

[2]Institute of Psychology, University of Würzburg, Würzburg, Germany.

[3]Institute of Human Biology, University of Hamburg, Hamburg, Germany.

*Corresponding Author:

Dr. Katharina A. Schwarz

Röntgenring 11

97070 Würzburg

Germany

+49-931-31-88655

katharina.schwarz@uni-wuerzburg.de

Table S3

Pain ratings and pain threshold temperatures (in °C) for each participant and day.

Subject	Group	Pain Ratings Day 1	Pain Ratings Day 2	Pain Threshold [°C] Day 1	Pain Threshold [°C] Day 2
1	MLPS	60.02	54.52	44.05	46.05
2	MLPS	57.78	26.27	42.10	45.18
3	MLPS	76.07	67.15	43.28	44.70
4	MLPS	69.22	49.90	44.60	45.20
5	MLPS	72.67	78.92	41.90	43.95
6	MLPS	67.03	64.47	41.45	43.93
7	MLPS	46.40	41.07	42.10	41.55
8	MLPS	69.53	72.72	45.53	46.10
9	MLPS	54.32	57.50	43.33	45.18
10	MLPS	54.53	44.52	45.30	46.38
11	MLPS	53.10	55.77	45.80	45.82
12	MLPS	53.28	56.18	37.45	37.33
13	MLPS	69.28	47.57	46.08	46.43
14	MLPS	71.40	49.62	45.15	46.83
15	MLPS	67.90	38.07	43.85	45.70
16	MLPS	68.48	60.00	45.15	45.95
17	MLPS	62.02	42.65	46.07	47.43
18	MLPS	73.78	61.96	43.55	42.68
19	MLPS	49.50	35.75	46.58	45.47
20	MLPS	73.04	57.38	41.78	43.58
21	MLPS	84.86	78.37	45.23	43.00
22	MLPS	91.65	69.77	44.58	45.22
23	MLPS	90.48	89.85	46.85	47.98
24	MLPS	68.08	74.48	38.90	39.15
25	MLPS	50.04	40.35	38.03	37.80
26	MLPS	60.15	60.28	43.25	47.18
27	MLPS	72.30	59.10	43.30	44.65
28	MLPS	71.13	85.60	37.95	48.68
29	MLPS	62.02	53.32	46.03	47.10
30	MLPS	56.92	52.00	42.10	45.13
31	MLPS	61.97	59.32	47.08	47.68
32	MLPS	46.48	8.53	46.05	47.35
33	MLPS	51.90	34.54	42.25	47.15
34	MLPS	71.26	55.47	45.98	47.00

35	FLPS	72.47	51.35	41.63	43.63
36	FLPS	72.42	68.98	44.80	43.10
37	FLPS	71.65	75.00	45.33	46.13
38	FLPS	53.86	59.28	41.73	43.23
39	FLPS	62.73	75.65	40.50	43.43
40	FLPS	66.65	76.20	43.78	43.23
41	FLPS	55.28	74.17	38.78	37.90
42	FLPS	53.82	55.83	46.98	47.40
43	FLPS	60.63	53.38	45.15	45.32
44	FLPS	42.10	73.67	41.43	43.10
45	FLPS	64.60	51.50	40.93	42.95
46	FLPS	68.42	71.70	40.75	42.70
47	FLPS	52.95	40.05	39.70	44.40
48	FLPS	58.73	31.89	39.15	37.60
49	FLPS	55.20	60.70	46.48	46.55
50	FLPS	56.78	60.60	47.83	46.93
51	FLPS	53.80	56.32	40.57	43.10
52	FLPS	63.38	85.07	38.93	39.53
53	FLPS	56.21	36.57	42.58	43.08
54	FLPS	64.22	50.56	47.30	47.95
55	FLPS	56.54	57.07	46.88	46.85
56	FLPS	81.82	60.31	47.38	46.88
57	FLPS	69.35	53.13	42.50	44.20
58	FLPS	69.02	61.21	48.28	48.15
59	FLPS	54.12	50.93	45.58	46.05
60	FLPS	39.96	46.29	41.40	44.68
61	FLPS	49.83	28.15	41.38	43.73
62	FLPS	54.14	36.28	42.13	40.38
63	FLPS	76.58	72.96	43.18	43.53
64	FLPS	73.80	76.62	39.43	39.20
65	FLPS	69.74	81.93	43.73	46.35
66	FLPS	95.13	85.99	44.30	45.58
67	FLPS	90.45	93.76	45.18	44.35
68	FLPS	64.17	54.13	44.78	42.32
69	FLPS	96.68	99.58	44.63	43.80
70	Control	79.75	79.18	46.50	44.90
71	Control	75.30	85.49	43.75	41.73
72	Control	35.91	38.91	38.40	39.13

73	Control	50.61	53.93	45.07	43.30
74	Control	59.62	73.79	42.58	42.40
75	Control	85.77	60.45	45.70	47.15
76	Control	96.92	88.77	47.75	47.40
77	Control	79.88	69.54	46.00	46.15
78	Control	85.18	61.20	45.10	43.45
79	Control	51.00	51.50	45.28	45.60
80	Control	70.65	61.50	44.38	43.35
81	Control	81.75	77.95	46.55	46.73
82	Control	84.75	82.00	46.08	45.38
83	Control	77.86	68.79	37.53	36.53
84	Control	46.98	41.17	41.75	39.73
85	Control	60.59	67.07	40.63	44.10
86	Control	61.80	69.32	39.60	40.75
87	Control	62.37	52.69	41.63	42.13
88	Control	66.82	64.87	44.53	39.65
89	Control	51.52	51.52	38.23	39.40
90	Control	62.08	53.83	38.45	40.50
91	Control	58.72	59.48	40.23	40.07
92	Control	61.78	68.63	41.73	41.38
93	Control	51.88	48.48	45.73	45.80
94	Control	60.73	58.91	41.65	40.70
95	Control	88.88	82.39	47.93	49.15
96	Control	87.23	76.82	43.55	45.15
97	Control	76.24	52.78	43.08	45.20
98	Control	76.30	54.97	43.80	46.40
99	Control	58.65	41.52	44.93	43.20
100	Control	74.37	50.25	44.03	40.45
101	Control	61.85	65.47	47.38	48.40
102	Control	70.14	36.01	46.45	46.33
103	Control	61.35	67.64	43.22	40.45
104	Control	71.23	57.28	38.58	38.50
105	Control	64.77	62.81	-	-

Table S4

Testosterone concentrations (TC; in pg/mL) for each participant in the fMRI experiment.

Subject	Group	TC [pg/mL]
1	MLPS	60.35
2	MLPS	140.38
3	FLPS	304.07
4	FLPS	212.08
5	FLPS	121.45
6	MLPS	96.47
7	MLPS	184.93
8	MLPS	123.23
9	MLPS	91.18
10	MLPS	144.75
11	MLPS	162.71
12	FLPS	145.59
13	FLPS	113.28
14	FLPS	96.80
15	FLPS	154.14
16	FLPS	276.31
17	FLPS	120.30
18	FLPS	143.28
19	FLPS	207.76
20	MLPS	122.49
21	MLPS	106.38
22	MLPS	140.53
23	MLPS	104.98
24	MLPS	161.23
25	MLPS	140.15
26	FLPS	115.59
27	FLPS	140.33
28	FLPS	91.44
29	MLPS	88.14
30	FLPS	83.38
31	MLPS	202.11

Table S5

Pain ratings and pain threshold temperatures (in °C) for each participant and day in the Naloxone experiment.

Subject	Group	Pain Ratings Day 1	Pain Ratings Day 2	Pain Threshold [°C] Day 1	Pain Threshold [°C] Day 2
1	Naloxone	51.39	65.13	44.30	44.28
2	Naloxone	74.64	67.11	46.10	45.53
3	Naloxone	53.10	35.61	40.80	45.15
4	Naloxone	78.91	63.82	41.75	40.10
5	Naloxone	72.45	60.30	44.18	45.50
6	Naloxone	45.59	44.78	46.53	46.73
7	Naloxone	77.12	52.35	42.08	46.45
8	Naloxone	48.37	40.36	47.53	46.97
9	Naloxone	73.93	71.52	44.30	43.63
10	Naloxone	73.93	53.32	44.15	44.33
11	Naloxone	75.37	66.28	38.88	37.68
12	Naloxone	55.08	37.38	38.58	42.90
13	Naloxone	62.78	58.98	45.40	44.05
14	Naloxone	81.63	76.30	46.77	47.00
15	Naloxone	47.60	38.68	40.95	40.82
16	Naloxone	76.81	74.39	43.95	45.38
17	Saline	80.74	72.46	45.18	44.85
18	Saline	56.48	53.10	41.03	44.93
19	Saline	78.47	55.89	38.85	37.70
20	Saline	71.44	67.52	43.18	43.58
21	Saline	53.84	48.18	39.55	43.80
22	Saline	83.24	62.79	44.88	44.88
23	Saline	73.60	63.75	44.50	45.43
24	Saline	56.40	42.94	37.93	40.13
25	Saline	48.25	52.38	39.33	41.13
26	Saline	74.78	54.01	42.85	39.72
27	Saline	59.94	33.66	42.18	43.88
28	Saline	42.28	28.93	42.58	46.03
29	Saline	60.30	58.22	46.28	46.68
30	Saline	91.45	73.56	46.68	47.20
31	Saline	78.42	58.78	47.63	48.18

Table S6

Pain ratings and pain tolerance measure (Pain Tol; in s) of the Cold Pressor Test (CPT) for each participant in the Cortisol experiment, and cortisol concentrations (CC; in µg/dL) for each participant and time point in the Cortisol experiment.

Subject	Group	CPT Pain Rating	CPT Pain Tol [s]	CC T1 [µg/dL]	CC T2 [µg/dL]	CC T3 [µg/dL]	CC T4 [µg/dL]	CC T5 [µg/dL]	CC T6 [µg/dL]
1	MLPS	91	56.69	.39	.29	.23	.30	.18	.25
2	MLPS	66	349.00	.25	.38	.28	.71	.47	1.12
3	MLPS	76	24.00	.59	.36	.25	.30	.23	.29
4	MLPS	92	25.94	.10	.08	.09	.10	.11	.11
5	MLPS	100	51.82	.52	.20	.14	.15	.10	.11
6	MLPS	84	69.04	.23	.22	.13	.22	.14	.13
7	MLPS	89	27.75	.45	.32	.32	.40	.36	.39
8	MLPS	95	83.31	.80	.31	.25	.21	.20	.68
9	MLPS	95	19.25	.12	.08	.06	.08	.06	.09
10	MLPS	100	95.00	.37	.16	.15	.25	.16	.14
11	MLPS	100	22.15	.26	.21	.15	.15	.18	.18
12	MLPS	68	59.00	.42	.27	.16	.21	.23	.18
13	MLPS	95	27.50	.25	.13	.12	.17	.15	.23
14	MLPS	99	25.34	.15	.15	.14	.26	.17	.18
15	MLPS	96	40.70	.42	.28	.29	.21	.29	.29
16	MLPS	39	310.85	.64	.31	.31	.81	.40	.50
17	MLPS	56	311.34	.23	.23	.19	.25	.18	.20
18	FLPS	100	33.16	.21	.12	.10	.09	.07	.04
19	FLPS	90	28.63	.31	.20	.17	.23	.13	.16
20	FLPS	96	51.19	.17	.12	.14	.29	.17	.16
21	FLPS	83	450.22	.26	.14	.14	.34	.24	.21
22	FLPS	100	19.50	.33	.11	.09	.54	.24	.19
23	FLPS	95	43.34	.79	.30	.23	.72	.49	.43
24	FLPS	85	25.66	.30	.22	.20	.50	.30	.28
25	FLPS	95	26.56	.53	.26	.13	.30	.18	.20
26	FLPS	88	20.84	.16	.27	.28	.15	.22	.17
27	FLPS	94	57.56	.26	.16	.10	.09	.06	.05
28	FLPS	100	32.47	.40	.19	.21	.46	.23	.23
29	FLPS	91	323.41	.22	.13	.13	.08	.07	.07
30	FLPS	99	19.00	.18	.30	.32	.14	.17	.18
31	FLPS	84	122.63	.23	.14	.23	.21	.21	.44
32	FLPS	100	67.06	.25	.16	.13	.27	.17	.15

33	FLPS	100	43.43	-	-	-	-	-	-
34	FLPS	96	45.28	.44	.72	.44	.18	.16	.12
35	FLPS	100	327.75	.18	.14	.13	.21	.21	.29
36	Control	72	147.00	.49	.24	.19	.28	.14	.16
37	Control	100	102.00	.26	.25	.26	.45	.24	.26
38	Control	100	23.41	.16	.06	.07	.09	.09	.04
39	Control	93	46.66	.36	.24	.20	.64	.40	.24
40	Control	94	17.22	.24	.13	.12	.28	.19	.16
41	Control	76	16.90	.23	.19	.19	.18	.23	.18
42	Control	93	15.19	.16	.13	.14	.16	.19	.24
43	Control	-	113.22	.07	.04	.03	.05	.06	.06
44	Control	84	246.78	.18	.17	.16	.10	.08	.11
45	Control	85	85.72	.37	.24	.14	.17	.18	.16
46	Control	80	188.37	.31	.27	.25	.21	.16	.14
47	Control	90	313.03	.38	.19	.16	.23	.13	.22
48	Control	97	41.81	.47	.24	.22	.36	.24	.25
49	Control	35	105.97	.36	.22	.21	.25	.29	.24
50	Control	100	41.75	.48	.35	.27	.54	.36	.64
51	Control	78	218.53	.49	.16	.19	.89	.36	.88
52	Control	100	59.50	.37	.21	.16	.23	.21	.14
53	Control	94	319.72	.26	.15	.23	.14	.17	.59
54	Control	20	327.19	.22	.75	.21	.22	.17	.53
